# Analysis of Wear Properties of Powder Metallurgy Steel in Abrasive Soil Mass

**DOI:** 10.3390/ma15196888

**Published:** 2022-10-04

**Authors:** Magdalena Lemecha, Jerzy Napiórkowski, Krzysztof Ligier, Wojciech Tarasiuk, Krzysztof Sztukowski

**Affiliations:** 1 Faculty of Technical Sciences, University of Warmia and Mazury in Olsztyn, 10-719 Olsztyn, Poland; 2 Faculty of Mechanical Engineering, Bialystok University of Technology, 15-351 Białystok, Poland; 3 Promostal, Fabryczna 7 Street, 16-020 Czarna Białostocka, Poland

**Keywords:** powder metallurgy steel, abrasive soil mass, wear of steel, spinning bowl method

## Abstract

This study presents the results of testing for abrasive wear of Vanadis 60 SuperClean powder metallurgy steel as compared to Hardox 600 steel and PMFe60P padding weld. The testing was conducted by the “rotating bowl” method using natural abrasive soil masses. Two types of abrasive masses with particle size distributions corresponding to light soil and medium soil were used. The obtained results enable the conclusion that the weight loss for Vanadis 60 SuperClean powder steel in both types of abrasive mass was approximately seven times lower than that for Hardox 600 steel and two times lower than PMFe60P padding weld. The high resistance of powder steel to abrasive wear in abrasive soil masses is related to the presence of a large number of fine M_6_C (tungsten-molybdenum) and MC (vanadium) carbide precipitates in its microstructure. The obtained test results indicate that the application of Vanadis 60 SuperClean steel may be extended to working elements operating in mineral abrasive environments.

## 1. Introduction

The content of alloy additions, particularly tungsten (W), vanadium (V), and chromium (Cr), promotes the formation of alloy carbides and allows high abrasion resistance to be achieved. Moreover, admixtures of these elements contribute to the formation of dispersive alloy carbide precipitates (M_4_C_3_, M_2_C, M_23_C_6_, M_6_C), which are responsible for the occurrence of the secondary hardness effect. Due to the low carbon concentration combined with the presence of elements such as chromium, tungsten, and silicon, alloy steels are characterised by higher ductility, which enables their use for the production of operating parts exposed to impact load [1,2,3,4,5,6,7].

The most common technologies for the deposition of layers containing a large proportion of these elements are welding methods such as hard facing. This treatment can be performed both in the production process and as part of operating part renovation. The service life of an operating part can be significantly prolonged in relation to the original material by selecting an appropriate material [8,9,10,11,12,13,14].

Many studies have described the effect of the addition of carbide-forming elements on changes in the structural and physicochemical properties of the surface layers of operating parts [15,16,17,18,19,20]. On this basis, it can be concluded that the operating part wear intensity is determined by the resistance of individual components of the particular material [19,21]. Not only are the properties of the material structure determined by the volume proportion of carbide precipitates but also by their dimensions, distribution, and shape [22,23,24]. The microscopic admixtures aimed to improve the properties of operating parts should be selected after analysing the conditions under which the particular tool will be operated and, thus, to what wear processes it will be exposed [25,26].

One of the technologies that enable the incorporation of alloying elements into steel is powder metallurgy. Powder metallurgy has found wide application in the production of sintered tool materials such as sintered metal carbides, carbide steel and high-speed steels with very high concentrations of carbon and alloying elements. This technology makes it possible to obtain materials with technological properties better than materials produced by conventional methods. In powder metallurgy (PM) steels, the carbide segregation and banding are nearly completely eliminated, even in products with the largest cross-sections.

An intermediate good for the production of sintered PM steels is a powder with a chemical composition corresponding to that of finished steel. The basic method for obtaining the powder involves the atomisation of liquid steel with inert gases (mainly nitrogen, occasionally argon or helium) or water. The obtained metal powders are mechanically pulverised and mixed in appropriate proportions to prepare them for further processing, possibly supplemented with additional elements.

The technological methods for producing PM steels can be divided into two groups [27,28]:

- Specialised or classic powder metallurgy methods that enable the production of ready tools or semi-finished products and products with a shape similar to the final shape;
- Combinations of powder metallurgy technology and conventional plastic forming to produce large-sized blocks, billets, or bars that are used to manufacture ready tools by machining methods.

The most widely used method for producing sintered steels involves the cold isostatic pressing of a steel powder compact, followed by its hot isostatic pressing sintering at a temperature of 1150 °C under a pressure of 100 MPa in an argon atmosphere. The alloy steel powder used in this method is obtained by atomising a regularly melted steel in a pure nitrogen stream. Steel compacts produced by this method exhibit a fine-grained and homogeneous structure in all areas. The sintered steel compacts are then subjected to soft annealing, after which they can be used directly to manufacture tools or are subjected to hot plastic forming to give the shape and dimensions most suitable for tool manufacture. Steels obtained by the sintering method are subjected to a similar heat treatment as conventional high-speed steels.

The studies into powder steels conducted to date and described in the literature are limited to the investigation of wear under the conditions of cutting tool operation [29,30,31].

Powder metallurgy steels, like Vanadis 60 SuperClear, are widely used for the production of industrial components operated under the conditions of sliding, rolling, or abrasive friction, e.g., gear wheels or cams. Vanadis 60 SuperClean is also used in the manufacture of cold work tools. Tests aimed at determining their tribological properties under the sliding friction conditions are described in many studies [32,33,34] and [35,36], which indicate that the processes of wear of the components produced by the powder metallurgy method are similar to those occurring in forged components under the same tribological conditions [37]. A study by Ceschini L. et al. [38] noted that the composition and sintering conditions had a considerable effect on the resistance of powder steels to sliding wear. In this regard, the best behaviour was noted for more hardenable Fe-C-Mo steels with a higher Mo content (1.5% *w/v*), sintered under conditions resulting in the formation of a bainite microstructure. On the other hand, in powder steels with the addition of vanadium, the dominant factor affecting the wear resistance is the size of the vanadium carbides [39].

The resistance of materials referred to as abrasive wear-resistant steels has been described in the literature in detail. These materials are categorised as specialty steels with such alloying elements as manganese and chromium, a precise, microscopic admixture of boron and with the addition of other alloying elements, e.g., molybdenum, nickel or titanium. A microscopic admixture of boron in these steels contributes to an increase in resistance to abrasive wear, which makes the materials of this type suitable for the production of operating parts of excavators, bulldozers, agricultural machines and tools, chutes and open load-carrying bodies, which has been confirmed by numerous studies [40,41]. A study by Kapcińska-Popowska [41], showed that Hardox 500 steel was characterized by good strength properties and resistance to abrasion, which makes it suitable for use in the manufacture of tools and operating parts of machinery. The appropriate characteristics of the steels of this type determine their wide use in construction, mining, and agriculture. A similar study was conducted by Napiórkowski [42,43], who compared the wear of five grades of abrasion-resisting steels in loamy sand with the wear of 38GSA steel used to produce agricultural tools.

Steels with boron additions are currently among the basic construction materials used for the production of machinery parts used under conditions of destructive abrasive environment impact [44,45,46]. Hardox 600 steel is a low-alloy steel intended for components exposed to abrasive wear. Steels of this type are characterized by high resistance to abrasive wear, machinability with specialized tools, weldability, high mechanical properties and resistance to impact load. The above-mentioned steel characteristics are achieved thanks to a precisely selected chemical composition and a low content of harmful admixtures (P and S).

PMFe60P is a metallic powder for plasma hardfacing used for the deposition of surface layers on components exposed to intensive abrasive wear in a mineral abrasive environment. PMFe60P is used for the production of hardfaced layers resistant to abrasion on soil-working elements, such as ploughs and the bucket teeth of excavators.

The tribological properties of powder steels indicate high resistance to general abrasive wear. To date, no information has been found on the possibility of the use of this type of steel under the conditions of being worn in the abrasive soil mass.

The aim of the study is to assess the wear intensity of powder metallurgy vanadium steel in two processing conditions in terms of the possibility of its use for working elements processing the abrasive mass of the soil.

## 2. Materials and Methods

### 2.1. Study Materials

The research was provided using the following materials: Vanadis 60 SuperClean (UDDEHOLMS AB, Hagfors Sweden) in two heat treatment states, Hardox 600 steel, and PMFe60P padding weld deposited onto 38GSA steel.

Vanadis 60 Super Clean steel is an alloy steel obtained by powder metallurgy (PM high-speed steel). It is a high-alloy, high-performance, high-speed powder steel with an admixture of cobalt. Vanadis 60 SuperClean is particularly suitable for cutting tools for which both high wear resistance and compression strength are required. The chemical composition, as declared by the manufacturer (UDDEHOLMS AB, Hagfors Sweden), is provided in Table 1.

The hardening process was carried out by heating at a temperature of 1100 °C for 5 min, followed by cooling at a rate of 10 °C/s. The cooling process was carried out in two variants, i.e., in a water bath and an oil bath. The hardening was carried out until a temperature of 25 °C was reached, followed by tempering at 560 °C. Three tempering cycles of 1 h each were carried out, with the specimens being cooled to room temperature between subsequent tempering cycles.

The analyses of Vanadis 60 SuperClean steel microstructure were conducted using a Phenom XL scanning electron microscope.

The declared by producer (SSAB) chemical composition of Hardox 600 steel is provided in Table 2.

The PMFe60P hardfaced layer was obtained by the plasma hardfacing method using a powder with a particle size distribution from 200 to 400 μm. The material was plasma-deposited on a 38GSA steel washer under an argon shield using an NP1-250-type device, (Welding Institute in Gliwice, Poland) with a current of 140 A, a hardfacing speed of 0.194 m/min and a powder consumption of 4.8 kg/h. The argon flow rate was set at 4.5 dm^3^/min. The produced padding weld was 6 mm thick and 8 mm wide. Single-layer hardfacing was applied.

The chemical composition of 38GSA steel declared by the manufacturer is presented in Table 3.

Table 4 presents the chemical composition of metallic powder used for hardfacing.

The declared chemical composition of the test materials was verified by the spectroscopic method using a Phenom XL scanning electron microscope (ThermoFisher Scientifis, Waltham, Massachusetts, Unitetd States) (SEM) with a backscattered electron detector (BSD), secondary electron detector (SE), and energy dispersive spectroscopy detector (EDS).

The hardness of the test materials was determined by the Vickers method in accordance with standard PN-EN ISO 6506 using a Wilson VH1150 (Buehler, Lake Bluff, IL, USA) hardness tester with a load of 98 N, which was maintained for 10 s.

The microstructure of Hardox 600 steel and PMFe60P padding weld was assessed using a Keyence VHX 7000 (KEYENCE INTERNATIONAL (BELGIUM) NV/SA, Mechelen, Belgium) digital optical microscope. The microstructure of Vanadis 60 SuperClean was assessed using a Phenom XL scanning electron microscope. The metallographic specimens were etched with Nital 5%.

### 2.2. Research Methodology

**Figure 1:**
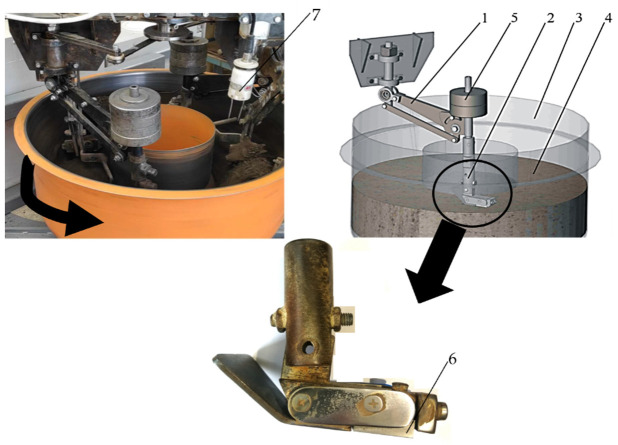
Test stand diagram: **1**—swingarm, **2**—specimen holder, **3**—bowl with abrasive mass (**4**), **5**—weight, **6**—specimen, **7**—moisture measure device.

The wear testing was carried out by the “rotating bowl” method (Figure 1) [47].

The test stand consisted of a bowl (3) filled with abrasive mass (4). Inside the bowl, there was a sample (6) mounted in a holder (2) suspended on the swingarm (1). The bowl was powered by an electric motor and made a rotary motion. The sample was pressed against the abrasive by the weight (5).

The sample holder was positioned to maintain a sample attacking angle of 7° (Figure 1). Such a positioning of the sample ensured the contact of the entire surface of the loaded sample with the abrasive mass throughout the entire test run.

The bowl was filled with natural abrasive soil mass of two types, defined as:

- A medium soil (sandy clay);
- A light soil (sandy loam).

The graining composition was assessed using a Mastersizer 2000+Hydro laser (Enigma Business Park, Malvern, United Kingdom) particle size analyser, while the moisture content of the soil was determined by measuring the weight of the solid phase dried at a temperature of 10 5°C. Abrasive mass moisture content was measured every 2000 m by Delta-T ML3 Theta Kit (AT Delta-T, Burwell, United Kingdom) (pos. 7 in Figure 1). During the tests, the moisture content was maintained at the level of 12% 

The particle size distribution of the soil masses is presented in Table 5.

The cuboid-shaped specimens with dimensions of 25 × 35 × 10 mm were cut out by the high-energy waterjet cutting method. In order to give the specimens the same surface conditions and final dimensions, they were processed by grinding.

The following testing parameters were applied:

- Sliding speed—1.7 m/s
- Sample load—49 N (load weight of 5 kg)
- Total sliding distance—20,000 m.

The test runs were repeated six times for each material in each of the abrasive masses.

Mass wear, expressed as weight loss, was adopted as a measure of wear. Mass wear was measured every 2000 m using a laboratory balance with an accuracy of 0.0001 g.

Mass wear was calculated from the relationship:

W_m_ = m_w_ − m_i_ (1)

where:

W_m_—mass wear (g);
m_w_—initial specimen weight before the wear testing (g);
m_i_—specimen weight after covering a specified friction distance (g).

The specimen surfaces after the wear test were assessed using a Keyence VHX7000 optical digital microscope.

## 3. Results

### 3.1. Materials Chemical Composition and Microstructure

**Figure 2:**
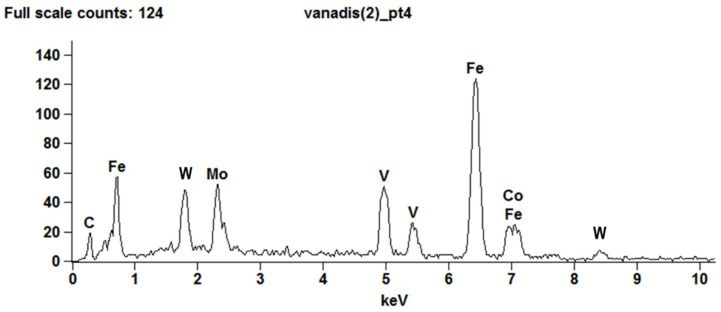
X-ray dispersive energy spectrum of the Vanadis 60 SuperClean steel.

The chemical composition of the Vanadis 60 SuperClean steel obtained by the spectrographic method did not differ significantly from the chemical composition declared by the manufacturer (Table 6). The X-ray dispersive energy spectrum (Figure 2) shows the presence of most of the chemical elements declared by the producer in the chemical composition.

**Figure 3:**
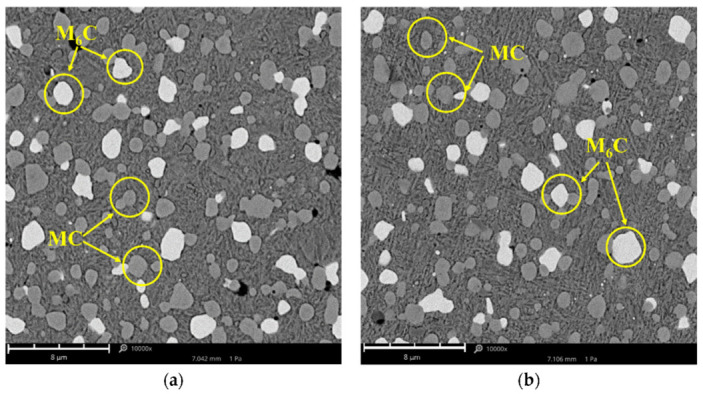
The microstructure of Vanadis 60 SuperClean steel hardened in oil (**a**) and in water (**b**). It was etched with Nital 5%. Scanning electron microscopy.

The presence in the chemical composition of W and Mo with a strong chemical affinity to carbon causes the formation of M_6_C carbides, and the addition of V creates very durable MC carbides (V_4_C_3_). The low chromium content (approx. 4%) causes it to completely dissolve in the iron, increasing the hardenability. On the other hand, Co is an additive that extends the range of austenite. Due to the high solubility in iron, it does not form carbides in steels. Vanadis 60 Super Clean steel was characterised by a homogeneous structure, which is typical of high-speed powder metallurgy steels. In this steel, the presence of M_6_C (tungsten-molybdenum—white) and MC (vanadium—dark grey) carbides was noted in the fine-grained structure of hardened martensite (Figure 3).

**Figure 4:**
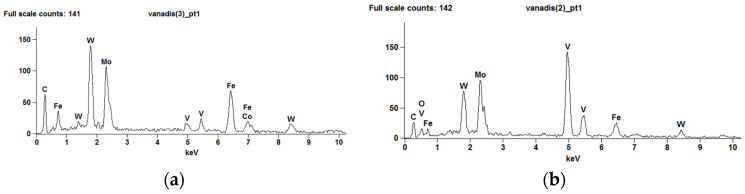
X-ray dispersive energy spectrum of the areas M_6_C (**a**) and MC (**b**) visible in Figure 3.

Figure 4 shows the energy spectrum of the carbide separation areas presented in Figure 3.

The increased content of W and Mo in the white areas, visible on the spectrum, indicates that these are carbides (W, Mo)_6_C, while the high content of V in the gray areas shows that they are carbides (V_4_C_3_).

The microstructure of Vanadis 60 Super Clean steel hardened in oil differed from that hardened in water in the number of carbide precipitates. The number of carbide precipitates was determined by the image analysis method using the Matlab Simulink 2020a program (MathWorks, Natick, MA, USA). For Vanadis 60 Super Clean steel hardened in oil, the proportion of all carbide phases on the surface was 25%, and for that hardened in water, it was 20%.

**Figure 5:**
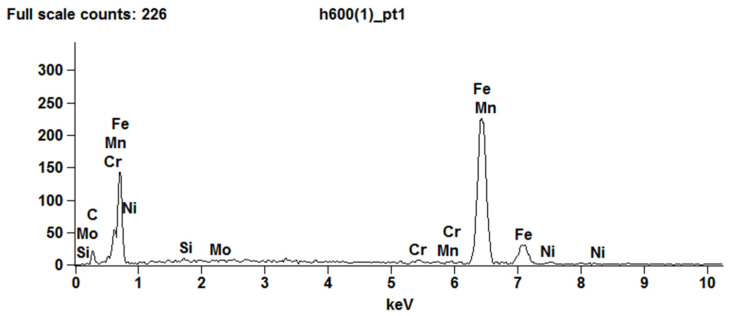
X-ray energy spectrum of Hardox 600 steel.

The spectrographic tests of the chemical composition of Hardox 600 (Table 7, Figure 5) steel showed a chemical composition similar to that declared by the manufacturer. Due to the low concentration of boron, it is not detectable by energy dispersion spectroscopy (EDS).

**Figure 6:**
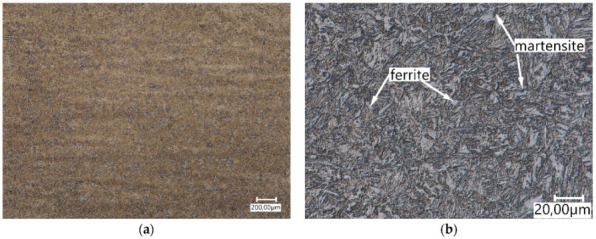
Hardox 600 steel microstructure (**a**) and magnified image of the microstructure (**b**). Light optical microscopy.

The low contains the elements Cr (approx. 1%) and Mn (approx. 1.2%), causing these elements to dissolve in iron without forming carbides, but only increasing the hardenability. Nickel (2.3%) present in Hardox 600 steel has an austenite-forming effect, and due to its good solubility in iron, it does not form carbides in steel. The microstructure of Hardox 600 steel was characterised by a banded structure resulting from rolling in the sheet production process (Figure 6a). The microstructure morphology indicates a martensitic structure with a varied orientation of martensite needles and plates (Figure 6b).

**Figure 7:**
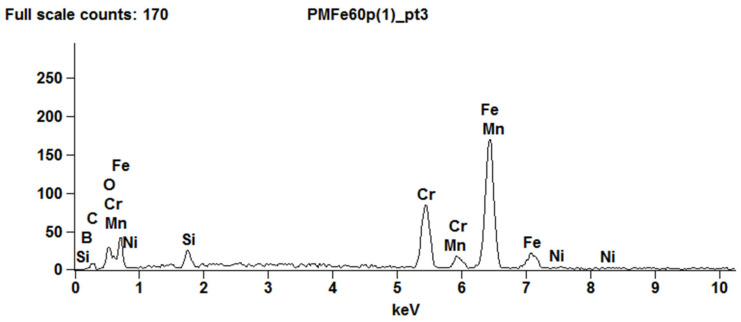
X-ray energy spectrum of PMFe60P padding weld.

The results of the spectrographic tests of the chemical composition of the PMFe60P padding weld (Table 8), (Figure 7) showed a 40% chromium content, which is a value higher than that declared by the manufacturer (approx. 33%). In such high concentrations (over 12%), chromium in steels shows a carbide-forming effect, forming M_7_C_3_ carbides. The composition also includes Mn and Ni, which are well soluble in iron, and which in steels do not form carbides at all (Ni) or in such low concentrations (Mn). High boron content reduces the hardenability of the steel and affects the growth of austenite grains.

**Figure 8:**
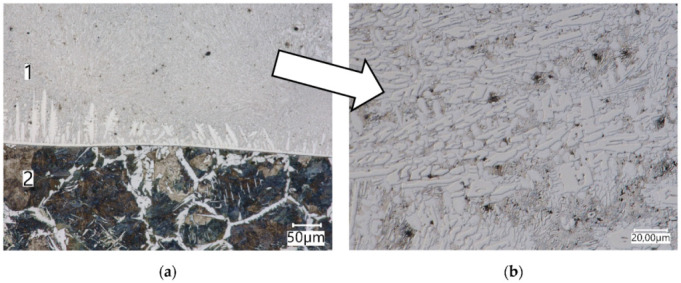
The microstructure of PMFe60P hardfaced layer; (**a**)—fusion zone; **1**—hardfaced layer, **2**—pad layer, (**b**)—hardfaced layer. Light microscopy.

Due to the high contents of Cr and Fe, after hardfacing, a microstructure of PMFe60P layer, consisting of alloy ferrite as well as M_7_C_3_ and MC carbides, was obtained. In the fusion penetration zone of the PMFe60P hardfaced layer, an alloy ferrite microstructure was identified, while in the padding weld, primary carbide precipitates (Fe, Cr)_7_ C_3_ with a lath structure in the eutectic matrix [α + (Fe, Cr)_7_C_3_], as well as borides and carbide/borides (Figure 8b), were identified.

### 3.2. Material Hardness Results

The hardness measurement results are provided in Table 9.

The highest hardness value was characterized by Vanadis 60 SuperClean steel, for which the lowest dispersion of the hardness values expressed as standard deviation was observed. This is related to the equal distribution of fine carbide precipitates in the microstructure. The greater hardness value dispersion of Hardox 600 steel can be explained by the banding of the microstructure resulting from the rolling of sheet metal in production. The highest dispersion of hardness values was observed for the PMFe60P padding weld. It is related to the presence of relatively large precipitates of chromium carbides, not very regularly distributed in the microstructure.

### 3.3. Wear Results

**Figure 9:**
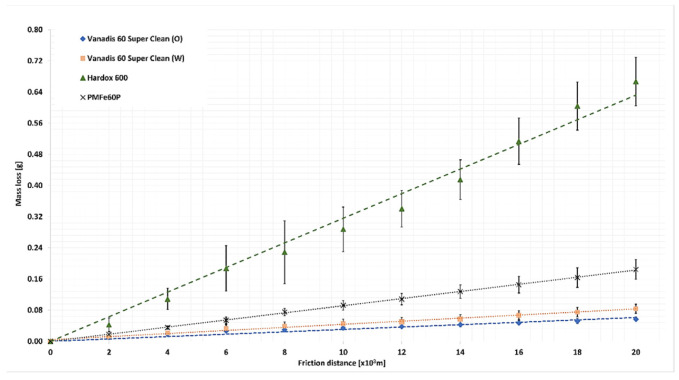
The course of weight loss for the test materials in the light soil.

**Figure 10:**
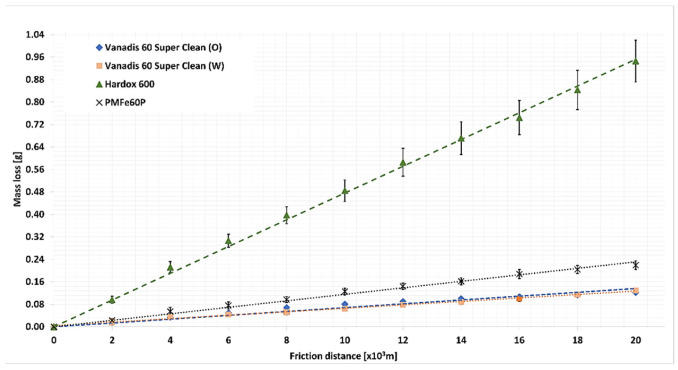
The course of weight loss for the test materials in the medium soil.

The course of weight loss for the test materials in the light abrasive mass is presented in Figure 9, while that in the medium abrasive mass is presented in Figure 10.

For all tested materials, the course of the loss of mass as a function of the friction path is linear. This means that the wear process is dominated by abrasive wear, such as drawing, ploughing, and micro-cutting. The highest weight loss (0.6667 g) was recorded for Hardox 600 steel, which is characterized by the lowest hardness among the tested materials. The less uniform wear pattern in light soil for Hardox 600 can be associated with the streaked structure of the microstructure. The most minor weight loss was recorded for Vanadis 60 SuperClean oil quenched (0.0565 g). This value is slightly lower than the weight loss value of this water-quenched material (0.0836 g). Vanadis 60 SuperClean steel in both tested machining conditions was worn approximately 12 times less than Hardox 600 steel. More than 3 times greater weight loss was recorded for the PMFe60P padding weld (0.1844 g), a material dedicated to application on surfaces subject to the most intensive wear of elements.

In the medium soil for all tested materials, the course of weight loss was also linear. In the case of this soil, also for Hardox 600 steel, no deviations of the wear pattern from a straight line were found. This may be related to strengthening the grains of the sandy fraction in the wet abrasive mass by the dusty fractions. Vanadis 60 SuperClean steel was characterized by the lowest weight loss in this type of soil. The difference in weight loss between water-hardened steel (0.1291 g) and oil (0.1215 g) was insignificant (Table 10). Comparing the weight loss of all tested materials, it can be concluded that for Hardox 600, the weight loss (0.9456 g) was 8 times greater, and for the PMFe60P padding weld, the weight loss (0.2194 g) was twice as high as for Vanadis 60 SuperClean steel.

Having compared the wear values for Vanadis 60 SuperClean steel in the abrasive masses used, it can be noted that the hardening method had a small effect on the weight loss value in the medium soil, while in the light soil, this effect is already distinct.

For Vanadis 60 SuperClean steel, the significance of differences in wear values was determined depending on the abrasive mass used. For this purpose, the ANOVA analysis of variance was performed. The results of mass wear after 20,000 m of friction distance were used for comparison. Then Duncan’s test was used as a posthoc test to distinguish homogeneous groups. The analysis was performed in Statistica 13.3 (StatSoft Kraków, Polnad). A null hypothesis about the lack of differences in the wear value and an alternative hypothesis about the occurrence of statistically significant differences were adopted. Where the null hypothesis was rejected in favour of the alternative one, the Duncan test was performed in order to distinguish significantly different groups. The results of variance analysis of the Vanadis 60 SuperClean steel wear value for the medium and light soil are provided in Table 10 and Table 11.

The variance analysis demonstrated that for the medium soil, the wear values were statistically insignificant, while for the light soil, the values were significant. Two groups with significant differences in wear were distinguished in this group (Table 12).

The differences in the wear value for Vanadis 60 SuperClean steel, depending on the abrasive mass type, can be attributed to the number of carbide phase precipitates depending on the cooling method. A greater number of carbide precipitates evenly distributed on the surface leaves less space for the soft matrix between them, which promotes wear resistance of the greater abrasive grains of the sand fraction. Larger abrasive grains which rest on hard carbide precipitates have no effect on the soft matrix. However, the small size of dust and silt grains allows them to penetrate between carbide precipitates and affect the relatively soft matrix. The light soil mass contains large amounts of sand fractions and small amounts of dust and silt fractions (Table 5), which is why significant differences in the wear of Vanadis 60 SuperClean, depending on the hardening method, occurred for this abrasive mass. A higher value of wear in the light soil was noted for Vanadis 60 SuperClean steel hardened in water, for which the percentage of the area of carbide precipitates is smaller by 5% than that for the steel hardened in oil.

### 3.4. Microscopic Analysis of Surfaces

**Figure 11:**
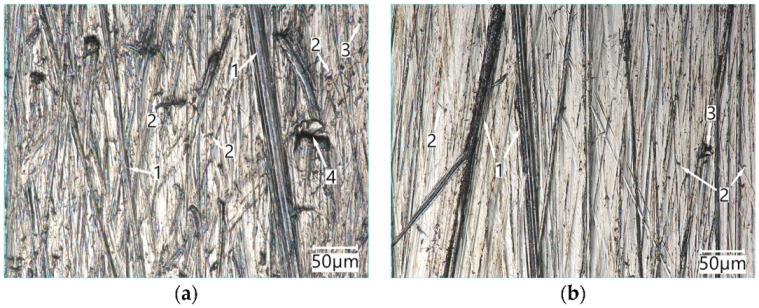
The surface of Hardox 600 steel worn in the medium soil (**a**) and light soil (**b**): **1**—ploughing, **2**—micro-cutting, **3**—chippings, **4**—plastic deformation. Optical light microscopy.

The surfaces of Hardox 600 steel specimens worn in the medium and light abrasive mass are presented in Figure 11.

The view of the surface of Hardox 600 steel worn in the medium soil (Figure 11a) shows considerable damage to the surface due to ploughing, micro-cutting and plastic deformation of the material. The surface of Hardox 600 steel worn in the light soil (Figure 11b) also shows traces of ploughing and micro-cutting, with the grooves and micro-cutting traces being shallower than those for the medium soil. This can be attributed to the greater freedom of abrasive grains, which are less consolidated with the silty fractions in a light abrasive soil mass than in a medium abrasive mass. As regards the homogeneous material (without carbide precipitates in the microstructure), i.e., Hardox 600 steel, the weight loss is primarily caused by the sand fraction abrasive grains.

**Figure 12:**
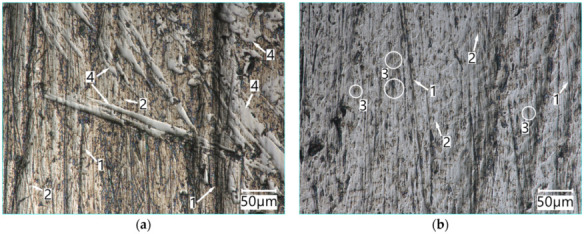
The surface of PMFe60P padding weld worn in the medium soil (**a**) and light soil (**b**): **1**—ploughing, **2**—micro-cutting, **3**—areas of material loss, **4**—carbide precipitates. Optical light microscopy.

The surface of the PMFe60P padding weld specimens after testing is presented in Figure 12. As regards the PMFe60P hardfaced layer, the presence of carbide precipitates in the microstructure resulted in less wear (Figure 12a).

**Figure 13:**
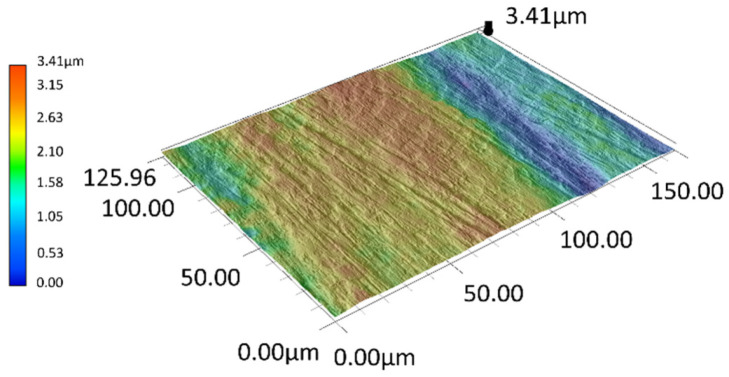
A surface profile of PMFe60P padding weld worn in the light soil. Optical light microscopy.

The surface of PMFe60P padding weld worn in the light soil shows shallow scratches resulting from ploughing and small traces of micro-cutting (Figure 12b). In addition, losses of the matrix material can be noted in the form of shallow oval depressions. The view of the surface suggests that the impact of loose abrasive grains resulted in the softer matrix phase being removed most intensively (Figure 13).

**Figure 14:**
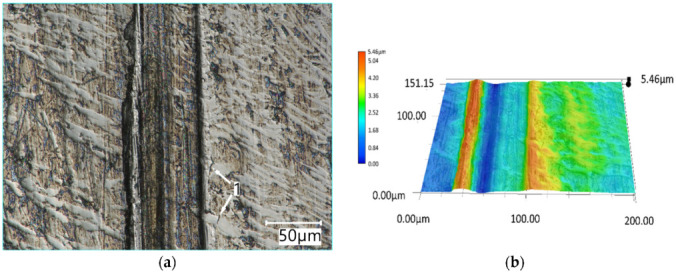
The surface of PMFe60P padding weld worn in the medium soil (**a**) and a surface profile of PMFe60P padding weld (**b**): **1**—cracks in the carbide phase at the groove boundary. Optical light microscopy.

The visible damage to the surface of the padding weld worn in the medium soil, resulting from ploughing and micro-cutting, concerns the area of the microstructure matrix. Large carbide precipitates resist these processes, provided that they are caused by single abrasive grains. On the other hand, the abrasive grains bonded by the silty fraction form aggregated soil particles whose impact is so strong that they are able to destroy large carbide precipitates. The trace of the impact of such a particle, as seen in Figure 14a, indicates the removal of the entire areas of carbide precipitates and the cracking of these precipitates at the boundary of the abrasive particle impact. The aggregated medium soil abrasive particles damage the surface more intensively by leaving deep grooves (Figure 14b).

**Figure 15:**
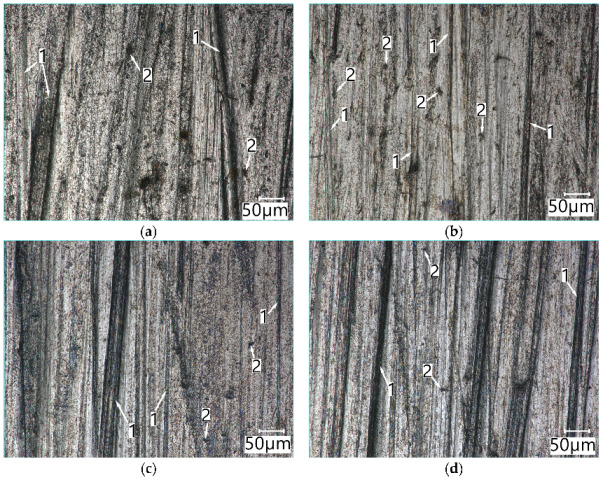
The surface of Vanadis 60 SuperClean (O) and Vanadis SuperClean (W) worn in the medium soil (**a**), (**c**) and light soil (**b**), (**d**): **1**—ploughing, **2**—chipping. Optical light microscopy.

The view of the surface of Vanadis 60 SuperClean steel (Figure 15) after being worn in the medium and light soil showed a large number of scratches resulting from ploughing. The difference in the view of the surface concerns the chipping marks found on the surface of specimens worn in the light soil (Figure 15b,d).

**Figure 16:**
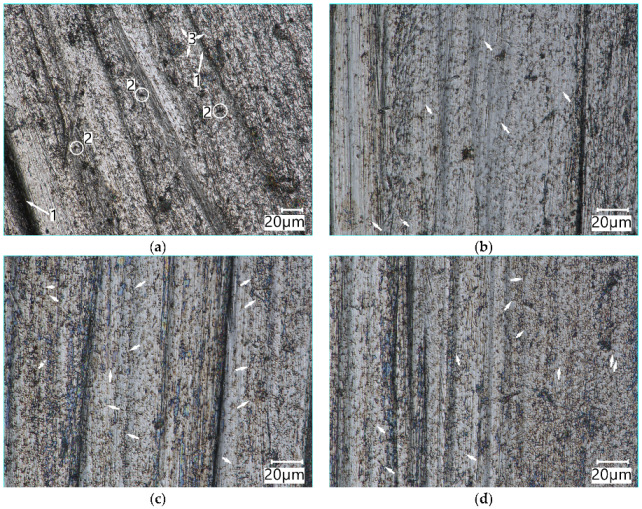
The surface of Vanadis 60 SuperClean steel (O) and Vanadis SuperClean (W) worn in the medium soil (**a**), (**c**) and light soil (**b**), (**d**): the arrows indicate selected carbide precipitates. **1**—ploughing, **2**—micro-cutting, **3**—fine carbide precipitates. Optical light microscopy.

The view of the surface of Vanadis 60 SuperClean steel (irrespective of the cooling method during the hardening process) indicates a highly homogeneous structure, similar to that of Hardox 600 steel. However, Vanadis steel was characterised by a several times lower weight loss. This is due to the presence of a large amount of fragmented molybdenum and vanadium carbides in its microstructure. These carbides, due to their uniform distribution in the microstructure, effectively resist the impact of both larger and smaller abrasive mass particles. The surface shows traces of ploughing and micro-cutting (Figure 16a), but they are shallow. Fine carbide precipitates protruding above the surface can be seen (Figure 16b). This can be seen on the surfaces of the specimens worn in the light soil (Figure 16b,d) and in the medium soil (Figure 16c).

The analysis of the surfaces reveals that the process of Vanadis 60 SuperClean steel wear proceeds similarly in both the light and the medium soil. The dust and silt fraction particles contained in both soil types have a wearing effect on the steel microstructure matrix area by removing small amounts of the material. Fine, hard M_6_C (tungsten-molybdenum) and MC (vanadium) carbides embedded densely in the microstructure resist the impact of larger abrasive grains of the sand fraction. The sand grains consolidated by silts and contained in the abrasive medium soil mass can remove larger portions of the material, thus resulting in larger scratches, as seen in Figure 16c.

## 4. Conclusions

1. Vanadis 60 SuperClean steel in both types of abrasive showed a much lower weight loss than Hardox 600 steel and PMFe60P padding weld. For light soil, the weight loss of Vanadis 60 SuperClean steel was approx. 12 times smaller than that of Hardox 600 steel and approximately 3 times lower than the PMFe60P padding weld. For medium soil, the weight loss was 8 times lower than that of Hardox 600 steel and 2 times lower than that of the PMFe60P padding weld.
2. Vanadis 60 SuperClean steel is characterised by a microstructure that contains a large number of fine molybdenum and vanadium carbide precipitates, which promotes resistance to wear in the fragmented abrasive medium environment.
3. Wear processes typical of abrasive wear, such as ploughing and micro-cutting, dominate in the tested materials. In materials with a different phase structure, a greater intensity of wear processes was observed in relation to the matrix, which resulted in the exposure of hard carbide phases. The exposed carbide phases effectively counteracted the wear processes, which, of course, lowered the consumption of Vanadis 60 SuperClean steel and PMFe60P padding welds compared to Hardox 600 steel.
4. The higher proportion of carbide precipitates on the surface of Vanadis 60 SuperClean steel hardened in oil promotes increased resistance to abrasive wear in light soil masses with a high content of sand fractions.
5. The obtained test results indicate that the application of Vanadis 60 SuperClean steel may be extended to working elements operating in mineral abrasive environments. The conducted tests should be treated as preliminary tests on the possibility of extending the application of Vanadis 60 SuperClean steel. Further research should be carried out to test the tribological properties of powder metallurgy steels in relation to the specific operating conditions of the working elements.

## Figures and Tables

**Table 1:** The declared chemical composition of Vanadis60 SuperClean steel.

Selected Chemical Elements (% *w*/*w*)					
C	Cr	Mo	W	V	Co
2.30	4.20	7.00	6.50	6.50	10.50

**Table 2:** The declared chemical composition of Hardox 600 steel.

Selected Chemical Elements [% *w*/*w*]								
C	Si	Mn	P	S	Cr	Ni	Mo	B
0.47	0.70	1.50	0.015	0.010	1.20	2.50	0.70	0.005

**Table 3:** The declared chemical composition of 38 GSA steel.

Selected Chemical Elements (% *w*/*w*)							
C	Si	Mn	P	S	Cr	Ni	Al
0.34 ÷ 0.42	0.8 ÷ 1.1	0.7 ÷ 1.1	max. 0.035	max. 0.040	max. 0.30	max. 0.3	max. 0.02 ÷ 0.06

**Table 4:** The declared chemical composition of PMFe60P metallic powder.

Selected Chemical Elements (% *w*/*w*)								
C	Si	Mn	Cr	Ni	Mo	B	Al	Cu
3.60	2.30	1.20	32.90	0.43	0.02	2.10	0.03	0.40

**Table 5:** Soil mass characteristics.

Soil Type	Sand 1 ÷ 0.1 mm (%)	Dust 0.1 ÷ 0.02 mm (%)	Suspended Fraction <0.02 mm (%)	Moisture Content (vol.) (%)
**Medium soil**	52.66	40.32	7.02	12.00
**Light soil**	77.48	20.83	1.69	12.00

**Table 6:** The declared chemical composition of Vanadis 60 SuperClean steel.

Selected Chemical Elements (% *w*/*w*)						
	C	Cr	Mo	W	V	Co
declared	2.30	4.20	7.00	6.50	6.50	10.50
author’s study	2.68	4.10	8.14	5.20	6.00	10.47

**Table 7:** The chemical composition of Hardox 600 steel.

Selected Chemical Elements (% *w*/*w*)									
	C	Si	Mn	P	S	Cr	Ni	Mo	B
declared	0.47	0.70	1.50	0.015	0.010	1.20	2.50	0.70	0.005
author’s study	0.43	0.24	1.00	-	-	0.95	2.30	0.80	-

**Table 8:** The chemical composition of PMFe60P padding weld.

Selected Chemical Elements (% *w*/*w*)									
	C	Si	Mn	Cr	Ni	Mo	B	Al	Cu
declared	3.60	2.30	1.20	32.90	0.43	0.02	2.10	0.03	0.40
author’s study	2.25	2.21	1.19	40.60	0.59	-	1.50	-	-

**Table 9:** Hardness measurement results of tested materials.

Materials	Vanadis 60 SuperClean W (Water-Hardened)	Vanadis 60 SuperClean O (Oil-Hardened)	Hardox 600	PMFe60P
Hardness [HV10]	961	938	635.5	784
Std. dev.	16.4	12.3	22.3	24.6

**Table 10:** The results of variance analysis of Vanadis 60 SuperClean steel wear value in the medium soil.

Subclass No	Duncan Test; Weight Loss Variable Approximate Probabilities for Post Hoc Tests Error: Intergroup MS = 0.00006, df = 10,000		
	Hardening bath type	0.1291	0.1215
1	W (water-hardened)		0.1116
2	O (oil-hardened)	0.1116	

**Table 11:** The results of variance analysis of Vanadis 60 SuperClean steel wear value in the light soil.

Subclass No	Duncan Test; Weight Loss Variable Approximate Probabilities for Post Hoc Tests Error: Intergroup MS = 0.00006, df = 10,000		
	Hardening bath type	0.0836	0.0565
1	W (water-hardened)		0.0011
2	O (oil-hardened)	0.0011	

**Table 12:** The results of the Duncan test for the wear value for Vanadis 60 SuperClean steel worn in light soil.

Subclass No	Duncan Test; Weight Loss Variable Approximate Probabilities for Post Hoc Tests Error: Intergroup MS = 0.00006, df = 10,000			
	Hardening bath type	Mean values	1	2
2	W (water hardened	0.0565	****	
1	O (oil hardened)	0.0836		****

****—illustrates the statistically significant difference *p* < 0.05 between the materials.

## Data Availability

All information and data are available within the articles.
